# Childhood trauma and mental health outcomes in Post-COVID Syndrome: Results from a cross-sectional study in Germany

**DOI:** 10.1016/j.bbih.2025.101069

**Published:** 2025-07-21

**Authors:** Andrea Stölting, Dominik Schröder, Tim Schmachtenberg, Inga Schimansky, Massa Yaqubi-Naqizadah, Christian Klemann, Franziska Rebmann, Marie Mikuteit, Sandra Steffens, Georg M.N. Behrens, Frank Klawonn, Alexandra Dopfer-Jablonka, Frank Müller, Christine Happle

**Affiliations:** a Department of Rheumatology and Immunology, Hannover Medical School, Hannover, Germany; b Department of General Practice University Medical Center Göttingen, Göttingen, Germany; c Department of Hematology, Hannover Medical School, Hannover, Germany; d Department of Pediatric Immunology, Rheumatology and Infectiology, Hospital for Childrens and Adolescents, University of Leipzig, Leipzig, Germany; e Department of Dermatology, Hannover Medical School, Hannover, Germany; f Deans' Office, Curricular Development, Hannover Medical School, Hannover, Germany; g German Center for Infection Research (DZIF), partner site Hannover-Braunschweig, Germany; h Department of Computer Science, Ostfalia University of Applied Sciences, Wolfenbüttel, Germany; i Biostatistics Research Group, Helmholtz Centre for Infection Research, Braunschweig, Germany; j Department of Family Medicine, Michigan State University, Grand Rapids, MI, USA; k Department of Pediatric Pneumology, Allergology, Neonatology, Hannover Medical School, Hannover, Germany; l German Center for Lung Research, DZL-BREATH, Hannover, Germany

## Abstract

**Introduction:**

Post-COVID syndrome (PCS) affects approximately 6–10 % of COVID-19 survivors, with complex clinical manifestations and poorly understood pathophysiological mechanisms. While childhood trauma is known to impact long-term health outcomes, its influence on PCS and associated quality of life remains unclear.

**Methods:**

In this cross-sectional study, we analyzed data from 641 participants (487 with PCS, 154 without) through the DEFEAT online platform. Childhood trauma was assessed using the Child Trauma Questionnaire-Short Form (CTQ-SF). Depression, anxiety, and health-related quality of life were evaluated using PHQ-9, GAD-7, and EQ-5D-3L. Uni- and multivariate analyses were performed.

**Results:**

Overall, 38.8 % of participants reported at least one adverse childhood experience (ACE). Emotional abuse was significantly more frequent in PCS patients compared to controls (21.1 % vs. 12.3 %, p = 0.02). PCS patients showed significantly higher rates of depression (18.9 % vs. 7.1 %, p < 0.001) and anxiety (23.8 % vs. 14.3 %, p = 0.004) compared to non-PCS participants. Health-related quality of life was significantly lower in PCS patients (median 64.9 vs. 90.2, p < 0.001). After adjusting for age, gender, and educational level, childhood trauma was associated with increased depression and anxiety specifically in PCS patients.

**Discussion/conclusion:**

Our findings suggest that childhood trauma significantly impacts mental health outcomes and quality of life in PCS patients. The strong association between childhood trauma and adverse mental health outcomes specifically in PCS patients highlights the importance of trauma-informed care approaches. These results emphasize the need for targeted screening and personalized interventions addressing both physiological and psychological aspects of PCS recovery in patients with childhood trauma history.

## 1 Introduction

According to conservative estimates, Post-COVID syndrome (PCS) affects an estimated 6–10 % of COVID-19 survivors and represents a significant global health burden (Global Burden of Disease Long et al., 2022; Thomas et al., 2024; Davis et al., 2023). It is a disease with thus far unclear definition and poorly understood pathophysiological mechanisms, typically associated with fatigue and cognitive, psychological, and physical impairments (Thomas et al., 2024). In the second half of 2023, around 250,000 out of 73 million publicly insured German persons received PCS related ambulatory care (Central Institute of Statutory Health Insurance Physicians Germany). In spite of the high demand for clinical care, factors leading to PCS are thus far unclear and treatment recommendations largely focus on symptom control (Lemhofer et al., 2024; Evidence reviews for signs and symptoms (update), 2021). PCS has manifold psychosocial and mental aspects, and the individual case histories for such factors, including childhood trauma, may significantly impact the risk for PCS (Martinez-Borba et al., 2023). Childhood trauma has a high global prevalence with 18–34 % of people reporting at least one adverse childhood experience (ACE) (Bellis et al., 2019). Childhood trauma fundamentally impacts long-term health outcomes, with affected individuals showing a significantly increased risk of cardiovascular disease (Alley et al., 2024), autoimmune conditions (Rehan et al., 2023), and mental health disorders independent of COVID-19 and PCS (Calegaro et al., 2023; Abate et al., 2024). The risk of experiencing a reduced health related quality of life (QoL) is vastly increased in persons with childhood trauma (Luo et al., 2024). While research has identified various risk factors for PCS development, emerging evidence suggests that adverse childhood trauma may play a crucial role in both susceptibility to and severity of long-term post-COVID symptoms, with persons reporting to have experienced ACEs carrying an estimated 1.4 fold increased risk for PCS (Vyas et al., 2024). Sustained psychological distress can lead to activation of the hypothalamic-pituitary-adrenal axis with subsequent immune dysregulation, which in turn can predispose for an increased PCS risk (Wang et al., 2022). A large-scale cohort study revealed a dose-dependent relationship between severity of childhood trauma and PCS severity with both sexual and physical/emotional abuse independently linked to PCS (Vyas et al., 2024). A trauma-informed perspective on PCS can have important implications for both clinical practice and public health policy, as targeted screening and personalized interventions could optimize the therapeutical approach to physiological and psychological aspects of PCS recovery. Understanding to what extent childhood trauma impacts the risk for PCS and QoL, and whether it is associated with a particularly poor QoL in affected patients is crucial for developing tailored treatment strategies and improving long-term outcomes for this vulnerable population.

## 2 Methods

### 2.1 Study design

To study the effect of childhood trauma in PCS in Germany, we performed cross-sectional data analyses based on the DEFEAT Corona (Defense Against COVID-19 Study, DOI: 10.2196/38718) study. The study protocol has been described in detail elsewhere (Mikuteit et al., 2022). DEFEAT prospectively analyses long-term consequences of COVID-19 and PCS. The study harbors an online platform (www.DEFEAT-corona.de) that offers information on PCS and provides access to electronic questionnaires and to interventional studies for persons with and without PCS. Thus far, more than 8000 people joined the DEFEAT platform. Participants are eligible to participate if they are (a) aged 18 years or older, (b) consent to participate, and (c) are proficient in the German language. After providing digital consent, participants are asked to complete a baseline questionnaire on their health status and receive follow up emails with links to surveys on their health status and additional research questions. In baseline questionnaires, we inquired information about sociodemographic characteristics, educational level, comorbidities, chronic diseases, SARS-CoV-2 infection and vaccination history, and possible onset and duration of PCS symptoms. For the current analyses, data from surveys completed between April 2022 and February 2023 were used. All results are reported according to the STROBE guidelines (von Elm et al., 2007).

### 2.2 Definition of PCS

PCS was defined for individuals reporting persistence or onset of new symptoms exceeding a four-week period after an acute SARS-CoV-2 infection confirmed by antibody, rapid-antigen (e.g. Lateral Flow Test), or PCR testing. Control participants were defined as persons that reported either to never have had COVID-19 or to have fully recovered from COVID-19 within four weeks post acute infection. Participants reporting an acute SARS-CoV-2 infection during the four weeks prior to childhood trauma assessment were excluded from the current analysis.

### 2.3 Assessment of childhood trauma

The short form Child Trauma Questionnaire (CTQ-SF) (Bernstein et al., 2003) was used to assess childhood trauma. The CTQ-SF is a self-reported 28-item inventory, which assesses severity and frequency of childhood trauma and explores adverse experiences in early life. The instrument comprises domains of emotional (≥13 points), sexual (≥8 points), physical abuse (≥10 points) and emotional (≥15 points) and physical (≥10 points) neglect. Items related to such ACEs can be checked using a 5-point Likert scale ranging from “never/none” (Global Burden of Disease Long et al., 2022) to “very often/severe” (Lemhofer et al., 2024). Cut off scores for the CTQ were based on Häuser et al., 2011 (Hauser et al., 2011). If at least one of the domains was above the cut off score, participants were categorized into having childhood trauma.

### 2.4 Assessment of participant characteristics

The analysis incorporated demographic variables including self-identified gender (male, female, non-binary), age (measured in years) and education (high [college preparatory qualification] or low [highest education below college preparatory qualification]). For psychological assessment and evaluation of health related quality of life (QoL), we utilized validated German versions of following instruments: the PHQ-9 for depression (Kroenke et al., 2001), GAD-7 for anxiety (Spitzer et al., 2006),and EQ-5D-3L for QoL (Schroder et al., 2024) Clinical significance was determined using established thresholds, for PHQ-9 scores exceeding 14 indicated moderate to severe depression, while GAD-7 scores above 9 suggested moderate to severe anxiety (Schroder et al., 2024).

### 2.5 Statistical analyses

Patients were excluded from analyses if the SARS-CoV-2 infection was acute or recent (<4 weeks) or had incomplete questionnaires regarding CTQ-SF, gender, age or education. Participant characteristics are presented as median (Q25-Q75) for numeric variables and as number of participants and proportion. When comparing PCS with No PCS participants Fisher's exact test or Wilcoxon-Mann-Whitney-Test was used for categorical or numerical variables, respectively. Regression analyses were conducted to explore the effects between health outcomes, CTQ and PCS status. When investigating binary variables as dependent variable (CTQ, PHQ-9 & GAD-7) a logistic regression model was used and when investigating hrQoL a linear regression model was used. All regression analyses were adjusted for gender, age and education. All statistical analyses were conducted using R (4.4.1), and Figures were plotted in Graphpad Prism (V4. GraphPad Software, La Jolla, CA/USA).

## 3 Ethics

The study protocol and data protection measures were approved by the responsible research ethics boards of Hannover Medical School (9948_BO_K_2021) and University Medical Center Göttingen (29/3/21). The study is registered in the German clinical trial registry (DRKS00026007) on September 9th^,^ 2021. All participants provided informed consent prior to study enrollment.

## 4 Results

**Fig. 1:**
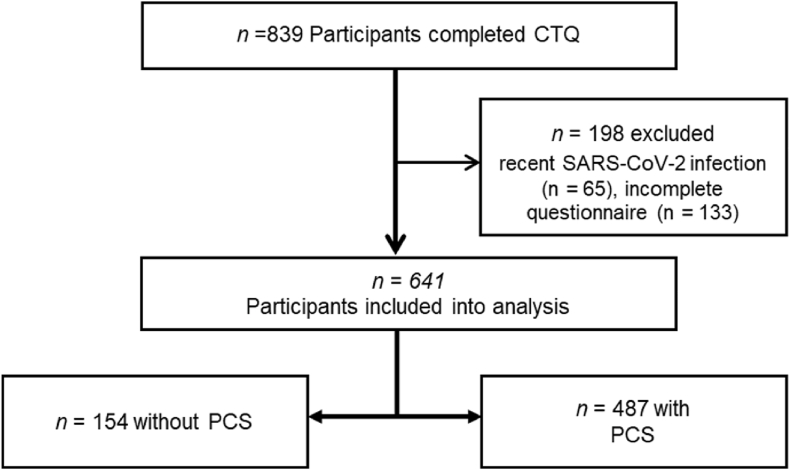
Flowchart of participant inclusion.

After applying the exclusion criteria, n = 641 subjects were included in the analyses (Fig. 1). Of these, n = 487 (76 %) met the definition criteria for PCS and n = 154 (24 %) did not report PCS-typical symptoms.

The median age of all respondents was 45 years (Q25-Q75 35–54, Table 1), and 78.1 % of them were female. None of the respondents identified as non-binary. Persons with PCS displayed approximately the same median age as those without PCS (43.7 years vs. 46.3 year), and a lower percentage of female participants occurred in the latter group (62.3 % vs. 83.2 %). Overall, 73.3 % of participants reported a high educational level, with a slightly higher fraction in non-PCS vs. PCS participants (86.4 % vs. 69.2 %).

**Table 1:** Demographic data and educational levels within the cohort.

	Total N = 641	No PCS *(n = 154)*	PCS *(n = 487)*
**Age** (median (Q25-Q75))	45.0 (35.0–54.0)	48.0 (36.0–57.0)	44.0 (34.5–53.0)
<40	253 (39.5)	58 (37.7)	195 (40)
40-60	343 (53.5)	75 (48.7)	268 (55)
>60	45 (7.0)	21 (13.6)	24 (4.9)
**Gender**			
Female	501 (78.1)	96 (62.3)	405 (83.2)
**Educational Level**			
High (College preparatory)	470 (73.3)	133 (86.4)	337 (69.2)

Data presented as n (%) if not other stated.

The frequencies of anxiety and depression were significantly higher in persons with PCS in comparison to those without, and their quality of life was significantly poorer (Table 2). The median anxiety score was 1.5 fold higher in respondents with PCS (p < 0.001), and depression scorings were even two fold increased in PCS vs. non PCS participants (p < 0.001, Table 2). Fittingly, PCS patient displayed a 1.7 fold higher prevalence of relevant anxiety, and a 2.7 fold increase in reporting of relevant depression (p = 0.004 and p < 0.001, respectively).

**Table 2:** Frequencies of mental pathologies in the cohort.

	Total N = 641	No PCS *(n = 154)*	PCS *(n = 487)*	p
**Anxiety (Median (Q25-Q75))**	5 (2–10)	4 (1–7)	6 (3–10)	<0.001^a^
**Anxiety >9 GAD-7 (n (%))**	138 (21.5)	22 (14.3)	116 (23.8)	0.004^2^
**Depression (Median (Q25-Q75))**	9 (5–13)	5 (2–8.3)	10 (7–14)	<0.001^a^
**Depression >14 PHQ-9 (n (%))**	103 (16.1)	11 (7.1)	92 (18.9)	<0.001^b^
**Health related QoL (Median (Q25-Q75))**	73.7 (61.3–90.2)	90.2 (75.2–100)	64.9 (48.4–79.4)	<0.001^a^
**No impairments EQ-5D-3L (n (%))**	111 (17.3)	74 (48.1)	37 (7.6)	<0.001^b^

a Wilcoxon-Mann-Whitney-Test.

b Fisher's exact test.

When assessing health related QoL, we observed significantly lower values in persons with PCS than in those without PCS (p < 0.001). No impairments using the EQ-5D-3L were reported by 17.3 % (n = 111) of all participants. When stratifying for PCS status, more than 6-fold more (48.1 %) of participants without PCS than those with PCS (7.6 %) reported no health related QoL impairments (p < 0.001).

Next, we analyzed the influence on childhood trauma on these factors. Overall, 249 (38.8 %) of all participants reported to have suffered from childhood trauma with at least one ACE in the fields of physical or emotional neglect, physical or emotional abuse, or sexual abuse (Table 3). For all ACE types, a higher proportion of participants with than without PCS reported to have suffered from at least one trauma event. The most prevalent ACEs occurred in the fields of emotional neglect and abuse, both within the entire cohort and in the population of participants with PCS, and emotional abuse was reported significantly more frequently amongst people with PCS than participants without this disease (1.7 fold more, p = 0.02, Table 3).

**Table 3:** Frequencies of childhood trauma and ACEs within the cohort.

	Total N = 641	No PCS (n = 154)	PCS (n = 487)	p^1^
**Childhood trauma (min. one scale)**	249 (38.8)	53 (34.4)	196 (40.2)	0.22
Emotional abuse	122 (19.0)	19 (12.3)	103 (21.1)	0.02
Emotional neglect	145 (22.6)	34 (22.1)	111 (22.8)	0.91
Physical abuse	48 (7.5)	11 (7.1)	37 (7.6)	1.00
Physical neglect	102 (15.9)	20 (12.9)	82 (16.8)	0.31
Sexual abuse	94 (14.7)	22 (14.3)	72 (14.8)	1.00

Data is presented as n (%); ^1^Fisher's exact test.

**Fig. 2:**
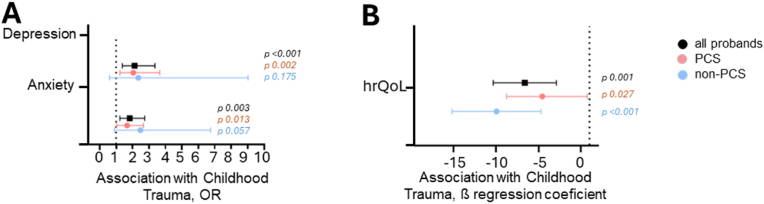
Impact of childhood trauma on anxiety, depression and health rela**ted QoL. A**: Childhood trauma dependent increase odds to report depression and anxiety. **B:** Association of childhood trauma with reduced health related QoL (Graph displays β regression coefficient with 95 % CI).

After adjustment for gender, age, and educational level, childhood trauma displayed a strong impact on depression, anxiety, and health related QoL across the entire cohort (Fig. 2). However, when comparing persons with and without PCS, the 95 % confidence intervals of the regression coefficients overlaped, indicating that the observed negative effect of childhood trauma on depression, anxiety and health related QoL was not statistically different between these groups.

## 5 Discussion

Post-COVID syndrome (PCS) affects hundreds of millions of people worldwide and is associated with significant morbidity and healthcare costs (WHO. Post COVID, 2024; Costantino et al., 2024; Sk Abd Razak et al., 2024). In spite of this enormous clinical need, PCS pathology is thus far largely unclear and treatment recommendations focus on symptom management (Davis et al., 2023; Lemhofer et al., 2024; Evidence reviews for signs and symptoms (update), 2021). The evidence that depression and anxiety are core phenomena and predictive factors of PCS is strong (Engelmann et al., 2024). Here, we show that childhood trauma is frequent in PCS, and that it significantly impacts anxiety, depression and health related QoL in affected patients. These pathologies harbor detrimental implications for a patient's participation in work, education, and social activities (Szewczyk et al., 2024).

In this context, risk stratification of patients is crucial in clinical decision making and ensuring they receive the medical aid and support they need. Our data shows that the experience of childhood trauma is frequent amongst PCS patients and suggests that information on trauma history can identify persons at particularly high risk for anxiety and depression, which have been suggested to be “core pathologies” of PCS (Engelmann et al., 2024; Masserini et al., 2024; Nicotra et al., 2023).

This may have important therapeutical implications as central nervous dysfunctions such as serotonin imbalances may play a central role in PCS pathology and open up new therapeutic angles (Wong et al., 2023; Butzin-Dozier et al., 2024). Although not yet sufficiently validated in clinical studies, preliminary analyses support the notion that treatment with specific serotonin reuptake inhibitors (10.13039/100017455SSRI) could alleviate 10.13039/100015534PCS symptoms (Mazza et al., 2022; Bonnet and Juckel, 2024), and also psychotherapeutic interventions such as cognitive behavioral therapy may lead to improvement of 10.13039/100015534PCS symptoms (Zeraatkar et al., 2024; Kuut et al., 2023).

In our cohort, childhood trauma, particularly emotional abuse, were reported more frequently by persons with PCS. This observation is in line with previous publications reporting on increased rates of childhood trauma among PCS patients (Martinez-Borba et al., 2023; Vyas et al., 2024). Importantly, we show that the combination of PCS and childhood trauma is associated with a particularly poor QoL, and that affected persons suffer from high rates of anxiety and depression, which illustrates the strong need for tailored treatment approaches.

The negative impact of childhood trauma on reduced health related QoL has been previously described independently of PCS (Luo et al., 2024). For PCS, this association has been described less clearly (Steinmetz et al., 2023). Our data shows that childhood trauma leads to a significant reduction of QoL in PCS, again advocating for patient tailored treatment approaches that could target pathologies related to this adverse event early in the patients lives.

Although our study is not powered to assess prevalences, the rate of childhood trauma in our cohort is comparable to frequencies described previously by other authors (Bellis et al., 2019). For Germany, a rate of 31.0 % of at least one ACE has been described (Witt et al., 2017), which is only slightly lower than the observed 38.8 % in our cohort. Also the pattern of ACE distribution with emotional abuse and emotional neglect being most frequently reported is in line with previous reports on childhood trauma in Germany (Kasinger et al., 2024). Childhood emotional abuse and emotional neglect have been described to be particular risk factors for depression in later life (Witt et al., 2017; Nelson et al., 2017). However, other surveys, e.g. the 2011–2020 Behavioral Risk Factor Surveillance System from the U.s. Center for Disease Control estimated the prevalence of at least one ACE among adults much higher (63.9 %) (Swedo et al., 2023). These strong differences in early trauma prevalences may be explained by distinct societal and cultural factors influencing parenting and the awareness for childhood abuse (Bellis et al., 2019; Levey et al., 2017), but may also be caused by different modes of data collection (Lemons et al., 2024).

Our study has important limitations. Firstly, as mentioned above, our sample size was limited, which may have prohibited us from detecting subtle effects of childhood trauma, particularly in the cohort on non-PCS patients. Due to this fact, we were able to only adjust for several potential confounders such as gender, age, and educational level. However, other studies with similar approaches but higher case numbers adjusted in the same fashion as we did (Klinger-Konig et al., 2024). Based on our study design, we cannot assess causality or the sequence of pathologies, e.g. to which degree depression, anxiety, or reduced QoL were present before development of PCS and how this impacted PCS occurrence and continuation. Also, we were unable to address implications of co-occurrence of multiple types of trauma within our cohort. Accumulating evidence suggest that mental distress before COVID-19 is a risk factor for PCS onset (Wang et al., 2022; Dempsey et al., 2024) and maintenance (Engelmann et al., 2024). Furthermore, it would have been desirable to assess PCS severity and to correlate this with the severity of childhood abuse as other authors did (Vyas et al., 2024). Such analyses will be focus of future research.

In spite of its limitations, our work has important implications. Our findings suggest that childhood trauma significantly impacts mental health outcomes and quality of life in PCS patients. The strong association between childhood trauma and adverse mental health outcomes specifically in PCS patients highlights the importance of trauma-informed care approaches. These results emphasize the need for targeted screening and personalized interventions addressing both physiological and psychological aspects of PCS recovery in patients with childhood trauma history.

## CRediT authorship contribution statement

**Andrea Stölting:** Writing – review & editing, Formal analysis, Writing – original draft. **Dominik Schröder:** Writing – review & editing, Methodology, Formal analysis, Writing – original draft, Investigation, Data curation. **Tim Schmachtenberg:** Writing – original draft, Writing – review & editing, Investigation. **Inga Schimansky:** Investigation, Writing – review & editing. **Massa Yaqubi-Naqizadah:** Investigation, Writing – review & editing. **Christian Klemann:** Investigation, Writing – original draft. **Franziska Rebmann:** Investigation, Writing – review & editing. **Marie Mikuteit:** Investigation, Writing – review & editing. **Sandra Steffens:** Writing – review & editing, Funding acquisition, Investigation. **Georg M.N. Behrens:** Investigation, Writing – review & editing, Funding acquisition. **Frank Klawonn:** Writing – review & editing, Formal analysis, Investigation, Conceptualization. **Alexandra Dopfer-Jablonka:** Writing – review & editing, Funding acquisition, Investigation. **Frank Müller:** Writing – original draft, Investigation, Formal analysis, Writing – review & editing, Methodology, Funding acquisition, Data curation. **Christine Happle:** Writing – original draft, Project administration, Funding acquisition, Conceptualization, Writing – review & editing, Supervision, Investigation, Formal analysis.

## Ethics approval and consent to participate

The Medical Ethics Committees of Hannover Medical School (9948_BO_K_2021) and University Medical Center Göttingen (29/3/21). The study is registered in the German clinical trial registry (DRKS00026007). All participants provided informed consent prior to study enrollment. For the prospective data analysis, all participants gave written informed consent.

## Consent for publication

This manuscript contains no individual patient data in any form.

## Availability of data and material

The data and biomaterials underlying our analysis may be requested by submitting a formal request to the study board, who will evaluate it. Email requests may be directed to happle.christine@mh-hannover.de.

## Declaration of generative AI and AI-assisted technologies in the writing process

During the preparation of this work C.H. used Claude.ai in order to check language, grammar and spelling, as well as structure of the paper. After using this tool, C.H. and all co-authors reviewed and edited the content as needed and take full responsibility for the content of the published article.

## Funding

This project is part of the DEFEAT-Corona Project funded by the European Regional Development Fund (ZW7-85152953). G.M.N.B. and A.D.-J. received funding (Niedersächsisches Ministerium für Wissenschaft und Kultur; 14-76103-184, COFONI 10.13039/100031212Network, project 4LZF23), G.M.N.B. was funded by the European Regional Development Fund ZW7-85151373, and A.D.-J. received funding by European Social Fund (ZAM5-87006761). C.H. received funding from the Excellence Cluster RESIST in infection research. D.S. was employed by 10.13039/100004319Pfizer Pharma GmbH when the manuscript was finalized and submitted. None of these sources had any role in the design and execution of the study, the writing of the manuscript and the decision to submit the manuscript for publication. The authors did not receive payment by a pharmaceutical company or other agency to write the publication. The authors were not precluded from accessing data in the study, and they accept responsibility for submission and publication of this article.

## Declaration of competing interest

D.S. was employed by Pfizer Pharma GmbH when the manuscript was drafted and submitted. No other author reports any competing interests.

## Data Availability

Data will be made available on request.
